# The molecular mechanism of action of superactive human leptin antagonist (SHLA) and quadruple leptin mutein Lan-2 on human ovarian epithelial cell lines

**DOI:** 10.1007/s00280-016-3113-8

**Published:** 2016-08-01

**Authors:** Elżbieta Fiedor, Ewa Łucja Gregoraszczuk

**Affiliations:** Department of Physiology and Toxicology of Reproduction, Chair of Animal Physiology, Institute of Zoology, Jagiellonian University, Gronostajowa 9, 30-387 Kraków, Poland

**Keywords:** Ovarian cell lines, ObR antagonists, Cell cycle, Signalling pathways

## Abstract

**Introduction:**

A number of leptin receptor antagonists have been synthesised for therapeutic use, with pre-clinical tests suggesting their future use in anticancer therapy. To our knowledge, there are no data concerning the possible application of leptin receptor blockers in ovarian cancer.

**Methods:**

In this study, we evaluated two leptin receptor antagonists: superactive human leptin antagonist (SHLA) and quadruple leptin mutein, Lan-2 (L39A/D40A/F41A/I42A), on cell proliferation (Alamar Blue test, BrdU assay), cell cycle gene (qPCR) and protein expression (Western blot) and cell signalling pathways (Western blot) in three different types of cell lines: OVCAR-3, CaOV-3 and HOSEpiC.

**Results:**

Both receptor blockers had no effect on non-cancerous HOSEpiC cell line proliferation; however, both reversed the stimulatory effect of leptin on CaOV-3 cell line proliferation to control levels and to below control levels in OVCAR-3 cells. In metastatic carcinoma CaOV-3, both ObR antagonists had an inhibitory effect on the cdk2/cyclin D1 complex, while in serous carcinoma, OVCAR-3, they only had an effect on cdk2 and cdk4 protein expression. SHLA had an inhibitory effect on all investigated signalling pathways in OVCAR-3, while only on Stat3 in CaOV-3. Lan-2 had an inhibitory effect on Stat3 and ERK1/2 in CaOV-3, while in OVCAR-3 it only affected Akt protein phosphorylation.

**Conclusion:**

Based on these results, we conclude that SHLA and Lan-2 are promising leptin receptor inhibitors which could be used to block leptin activity, eliminating its negative effects on activities related to carcinogenesis. However, the selection of a specific antagonist should be related to tumour type.

## Introduction

Leptin is a small (16-kDa) protein produced and secreted by adipose tissue, which is involved in appetite regulation, bone formation and reproductive function. Recent studies indicate that leptin via stimulatory action on cell proliferation, apoptosis and angiogenesis can promote an aggressive cancer [1–3]. Epidemiological studies have suggested a positive correlation between obesity and an increased risk of different cancers [4]. Serum leptin levels have been reported to be higher in overweight and obese women than in normal-weight women [5]. The normal level of leptin is about 4 ng/mL, in obese people a tenfold higher concentration has been noted, and in extremely obese patients it can range up to 100 ng/mL [6]. Cancer risk is higher among overweight (about 16 %) and obese (about 30 %) people [7]. Uddin et al. [8] revealed a significant association between leptin receptor (ObR) overexpression and poor survival rates in 59.2 % of epithelial ovarian cancers. Leptin and its receptors are overexpressed in different human cancers [9]. It has been proposed as a marker of prostate, breast and oesophageal cancer [10, 11] and one of the six markers of ovarian cancer [12].

Ptak et al. [2], using an OVCAR-3 cell line, showed that leptin promotes cell line growth by upregulating genes and proteins responsible for inducing cell proliferation, as well as downregulating proapoptotic genes and proteins in apoptotic pathways. Chen et al. [13] demonstrated that leptin upregulates the expression of cyclin D1 and Mcl-1, stimulating cell growth by activating the PI3 K/Akt and MEK/ERK1/2 pathways in the OVCAR-3 cell line. Cuello-Fredes et al. [3] showed serum and ascites leptin levels were significantly higher in overweight patients with worse survival. Moreover, they found worse overall survival in patients expressing higher leptin/ObRb mRNA levels.

Currently several groups of scientists are working on the synthesis of molecules that block the leptin receptor (ObR). A number of leptin receptor antagonists have been synthesised for therapeutic use, with several completing pre-clinical testing [14], suggesting their future use in anticancer therapy. The leptin antagonists Aca-1 and Allo-aca inhibit leptin-stimulated proliferation in breast cancer cells, MCF-7 and MDA-MB-23 [15]. Another leptin antagonist LDFI (leptin binding site I) also inhibits leptin-stimulated proliferation of breast cancer cells, MCF-7 and SKBR-3 [16]. Lan-1 has been shown to inhibit leptin signalling in the prostate cancer cell line LNCaP [17]. Bain et al. [18] have shown that superactive human leptin antagonist (SHLA) alone or in combination with cisplatin is a potential anticancer agent in gastro-oesophageal adenocarcinomas. Our previous work has shown that SHLA can reverse leptin-induced hormone secretion in porcine ovarian follicles [19]. To our knowledge, there are currently no data concerning the possible application of leptin receptor blockers in epithelial ovarian cancer.

Epithelial ovarian cancers are classified based on their cell type, serous (30–70 %), endometrioid (10–20 %), mucinous (5–20 %), clear cell (3–10 %) or undifferentiated (1 %). Therefore, we used four ovarian cancer cell lines isolated from different histological carcinomas: OVCAR-3 serous carcinoma, SKOV-3 TNF-resistant carcinoma, CaOV-3 metastatic carcinoma and TOV-21G clear-cell carcinoma. The cell lines were exposed to two leptin antagonists: superactive human leptin antagonist (SHLA) and quadruple leptin mutein Lan-2 (L39A/D40A/F41A/I42A), which bind to leptin receptors but do not activate them.

We tested the antagonists action on the expression of the cell cycle, apoptosis-related genes, proteins and the standard pathways of action of anticancer cell lines, with the highest receptor expression compared to action in non-cancerous human ovarian surface epithelial cells (HOSEpiC).

## Materials and methods

### Reagents

Ovarian Epithelial Cell Medium (OEpiCM) was obtained from Scintila (Jihlava, Czech Republic). McCoy’s 5a Medium Modified, Dulbecco’s Modified Eagle’s Medium (DMEM), RPMI-1640, MCDB 105 Medium and Medium 199, foetal bovine serum (FBS, heat inactivated), penicillin and streptomycin were obtained from the Sigma Chemical Co. (St. Louis, MO, USA). Leptin was obtained from the Sigma Chemical Co. (St. Louis, MO, USA). Leptin receptor antagonists were obtained from Protein Laboratories Rehovot (PLR) Ltd. (Rehovot, Israel).

### Cell culture

Human Ovarian Surface Epithelial Cells (HOSEpiC), (Scintila, Jihlava, Czech Republic) were cultured in OEpiCM. Human ovarian epithelial carcinoma cell lines, OVCAR-3, CaOV-3, SK-OV-3 and TOV-21G, were obtained from the American Type Culture Collection (Manassas, VA, USA). Cells were routinely cultured in RPMI-1640 supplemented with 20 % FBS, DMEM with 10 % FBS, McCoy’s 5a medium supplemented with 10 % FBS and in a 1:1 mixture of medium 199 and MCBD 105 medium with 15 % FBS, respectively. All media were supplemented with 50 IU/mL of penicillin and 50 μg of streptomycin. Cells were grown in 75 cm^2^ tissue culture dishes (Nunc, Denmark) in a 37 °C incubator with a humidified mixture of 5 % CO_2_:95 % air.

### qPCR analysis

Assuming the expression of the gene for the leptin receptor in cancerous and non-cancerous human ovarian epithelial cell line and action of leptin on ObR gene expression, cells were seeded into 96-well culture plates at a density of 1 × 10^4^ cells/well HOSEpiC, 1 × 10^4^ cells/well SK-OV-3, 1.5 × 10^4^ cells/well TOV-21G, 1.5 × 10^4^ cells/well CaOV-3 and 1 × 10^4^ cells/well OVCAR-3. The next day the medium was changed and the cells were treated with leptin at two doses, 40 ng/mL and 100 ng/mL, for 24 h. Total RNA isolation and cDNA synthesis were performed using the TaqMan Gene Expression Cell-to-CT Kit (Applied Biosystems, Carlsbad, CA, USA) following the manufacturer’s protocol. Amplifications were performed using the StepOnePlus system (Applied Biosystems, Carlsbad, CA, USA) and the TaqMan Leptin Receptor starter (Cat. No. Hs00174497_m1) in combination with the TaqMan Gene Expression Master Mix (Applied Biosystems, Carlsbad, CA, USA), following the manufacturer’s instructions. A PCR was performed using a final volume of 20 μL, including 50 ng/reaction cDNA.

In experiments with antagonists, cells were seeded into 96-well culture plates at a density of 1.5 × 10^4^ cells/well CaOV-3 and 1 × 10^4^ cells/well OVCAR-3. The next day the medium was changed and the cells were treated with leptin at a dose of 40 ng/mL alone or with SHLA or Lan2 at a dose of 1000 ng/mL for 24 h. cDNA synthesis was performed using the TaqMan Gene Expression Cell-to-CT Kit (Applied Biosystems, Carlsbad, CA, USA) following the manufacturer’s protocol. Amplifications were performed using the StepOnePlus system (Applied Biosystems, Carlsbad, CA, USA) and the TaqMan Array, Human Cyclins and Cell Cycle Regulation, Fast 96-well (Cat. No. 4418768) in combination with the TaqMan Gene Expression Master Mix (Applied Biosystems, Carlsbad, CA, USA), following the manufacturer’s instructions. A PCR was performed with a final volume of 10 μL including 50 ng/reaction cDNA.

The PCR conditions were as follows: pre-incubation for 2 min at 50 °C and 10 min at 95 °C, amplification for 40 cycles (15 s at 95 °C and 1 min at 60 °C). The relative expression of genes was normalised against the endogenous reference gene GAPDH (Human GAPD Endogenous Control, number 4333764F) (ΔC_q_) and converted to relative expression (RQ) using the 2^−ΔΔCq^ method. The results are expressed in the figures as relative values (RQ).

### Cell proliferation BrdU assay

DNA synthesis in proliferating cells was determined by measuring bromodeoxyuridine (BrdU) incorporation with the commercial Cell Proliferation ELISA System (Roche Molecular Biochemicals, Mannheim, Germany). The cells were seeded in 96-well culture plates at a density of 1 × 10^4^ cells/well HOSEpiC, 1 × 10^4^ cells/well CaOV-3 and 0.8 × 10^4^ cells/well OVCAR-3. Leptin was added at dose of 40 ng/mL. Leptin receptor antagonists were added at doses of 10, 100, 1000 ng/mL, with leptin at a dose of 40 ng/mL. Cells were cultured for 72 h with repeated exposure (the culture medium was changed every day and fresh compounds were added). Afterwards, the medium was removed and the cells were incubated for 3 h with a BrdU labelling solution (provided by the kit) containing 10 µM BrdU. The assay was performed following the manufacturer’s instructions. Absorbance values were measured at 450 nm using an ELISA reader (ELx808 BIO-TEK Instruments, Vinooski, VT, USA). Culture medium alone was used as a control for non-specific binding.

### Cell proliferation Alamar Blue assay

HOSEpiC cells were seeded in 96-well culture plates at a density of 6 × 10^3^ cells per well, OVCAR-3 cells were seeded at a density of 7 × 10^3^ cells per well, and CaOV-3 cells were seeded at a density of 6 × 10^3^ cells per well. Leptin receptor antagonists were added at doses of 10, 100, 1000 ng/mL, with leptin at a dose of 40 ng/mL. Cells were cultured for 72 h with repeated exposure (the culture medium was changed every day and fresh compounds were added). After 72 h, Alamar Blue stock solution was aseptically added to the wells in amounts equal to 10 % of the incubation volume and incubated for 4 h with the cells; the assay was performed following the manufacturer’s instructions. The resazurin reduction was measured at a 540 nm excitation wavelength and a 590 nm emission wavelength using a FLUORO-microplate reader (BIO-TECH Instruments, USA).

### Western blot analysis

Cells were plated into 24-well plates at a density of 10 × 10^4^ cells for OVCAR-3 cells, for CaOV-3 and 8 × 10^4^ cells HOSEpiC cells and allowed to attach overnight. The next day the media were changed and the cells were treated with 40 μg/mL leptin alone or in combination with 1000 μg/mL SHLA or Lan-2. To examine cell cycle protein expression, cells were incubated for 72 h (OVCAR-3) or 48 h (CaOV-3, HOSEpiC). After incubation, the cells were washed with ice-cold PBS and lysed with Laemmli lysis buffer (Sigma Chemical Co., St. Louis, MO, USA). The lysed cells were then scraped, transferred to microtubes and stored at −70 °C until analysis.

Before analysis, samples were sonicated and centrifuged at 15,000 × *g* for 15 min at 4 °C. The quantity of proteins was determined using the Bradford method, and the clear supernatant was used for electrophoresis. Equal amounts of protein (100 µg) from each treatment group were separated by SDS-PAGE and transferred to PVDF membranes using a Bio-Rad Mini-Protean 3 apparatus (Bio-Rad Laboratories Inc., Hercules, CA, USA). The blots were blocked for 1 h in 5 % BSA with 0.1 % Tween-20 in 0.02 M TBS buffer. Blots were incubated overnight with primary antibodies specific to ObR (ab5593, abcam, Cambridge, Great Britain; dilution 1:2000). After incubation with the primary antibody, the membranes were washed three times with 0.1 % Tween-20 in 0.02 M TBS buffer and incubated for 1 h with an appropriate horseradish peroxidase-conjugated secondary antibody (#7074, Cell Signaling Technology Inc., Beverly, MA, USA; dilution 1:2000).

β-Actin was used as an internal loading control; membranes were washed for 30 min in stripping buffer (0.25 M glycine, 1 % SDS, pH 2) and reprobed by overnight incubation with primary antibodies specific to β-actin (A5316, Sigma Chemical Co., St. Louis, MO, USA; dilution 1:2000) and for 1 h with a horseradish peroxidase-conjugated secondary antibody (P0447 DAKO, Glostrup, Denmark; dilution 1:5000).

Immunopositive bands were visualised using Western Blotting Luminol Reagent (Santa Cruz Biotechnology Inc., Santa Cruz, CA, USA) and ChemiDoc™ XRS+System (Bio-Rad Laboratories Inc., Hercules, CA, USA). The relative levels of protein expression were determined using ImageJ software (US National Institutes of Health, Bethesda, MD, USA). Individual protein levels were normalised to β-actin controls, and the ratio of protein to β-actin was normalised to 1 in the untreated control group.

To study cell cycle protein level cells were plated into 24-well plates at a density of 10 × 10^4^ cells for OVCAR-3 cells and 9 × 10^4^ cells for CaOV-3 cells and allowed to attach overnight. The next day the media were changed and the cells were treated with 40 μg/mL leptin alone or in combination with 1000 μg/mL SHLA or Lan-2. To examine cell cycle protein expression, cells were incubated for 72 h (OVCAR-3) or 48 h (CaOV-3). Equal amounts of protein (60 µg) from each treatment group were separated by SDS-PAGE and transferred to PVDF membranes. The blots were blocked for 1 h in 5 % BSA with 0.1 % Tween-20 in 0.02 M TBS buffer. Blots were incubated overnight with primary antibodies specific to Cyclin D1 (#2978, Cell Signaling Technology Inc., Beverly, MA, USA), cdk4 (#12790), cdk2 (#2546), cyclin A2 (#4656) at a 1:1000 dilution and E2F-2 (sc-251 Santa Cruz Biotechnology Inc., Santa Cruz, CA, USA) at a dilution of 1:200. After incubation with the primary antibody, the membranes were washed three times with 0.1 % Tween-20 in 0.02 M TBS buffer and incubated for 1 h with an appropriate horseradish peroxidase-conjugated secondary antibody (#7074, Cell Signaling Technology Inc., Beverly, MA, USA; dilution 1:2000 and sc-2005, Santa Cruz Biotechnology Inc., Santa Cruz, CA, USA, respectively).

GAPDH was used as an internal loading control; membranes were washed for 30 min in stripping buffer (0.25 M glycine, 1 % SDS, pH 2) and reprobed by overnight incubation with primary antibodies specific to GAPDH (G-8795, Sigma Chemical Co., St. Louis, MO, USA; dilution 1:20 000) and for 1 h with a horseradish peroxidase-conjugated secondary antibody (sc-2005, Santa Cruz Biotechnology Inc., Santa Cruz, CA, USA dilution 1:2000).

Immunopositive bands were visualised using Western Blotting Luminol Reagent (Santa Cruz Biotechnology Inc., Santa Cruz, CA, USA) and ChemiDoc™ XRS+System (Bio-Rad Laboratories Inc., Hercules, CA, USA). The relative levels of protein expression were determined using ImageJ software (US National Institutes of Health, Bethesda, MD, USA). Individual protein levels were normalised to GAPDH controls and the ratio of protein to GAPDH was normalised to 1 in the untreated control group.

To study leptin receptor signalling, the cells were treated with 40 ng/mL of leptin in combination with SHLA or Lan-2 at a concentration of 1000 μg/mL for 0, 5, 15, 30 and 60 min. Sixty micrograms of protein from each treatment group was separated by 10 % SDS-PAGE. Blots were incubated overnight at 4 °C with antibodies specific for phospho-Stat3 (Tyr705) (#9131), Stat3 (#9132), phospho-p44/42 MAPK (#9101), p44/42 MAPK (#9102), phospho-Akt (Ser473) (#9271) and Akt (#9272) at a dilution of 1:1000 (Cell Signaling Technology Inc., Beverly, MA, USA). After incubation with the primary antibody, the membranes were washed three times and incubated for 1 h with a horseradish peroxidase-conjugated secondary antibody (#7074) at a dilution of 1:2000 (Cell Signaling Technology Inc., Beverly, MA, USA). Immunopositive bands were visualised using Western Blotting Luminol Reagent (Santa Cruz Biotechnology Inc., Santa Cruz, CA, USA) and ChemiDoc™ XRS+System (Bio-Rad Laboratories Inc., Hercules, CA, USA). The relative levels of protein expression were determined using ImageJ software (US National Institutes of Health, Bethesda, MD, USA). Individual protein levels were normalised to the total amounts of signalling proteins and the ratio of phospho-protein to total protein was normalised to 1 in the untreated control group (time = 0 min).

### Statistical analysis

Data were expressed as mean ± SEM from the four independent experiments performed in triplicate. Statistical analyses were performed using GraphPad Prism 5. Data were analysed using a one-way analysis of variance (ANOVA) followed by a Tukey’s honestly significant difference (HSD) multiple range test. A value of *p* < 0.05 was considered statistically significant.

## Results

### The expression of the leptin receptor (ObR) gene in different cell lines

**Fig. 1:**
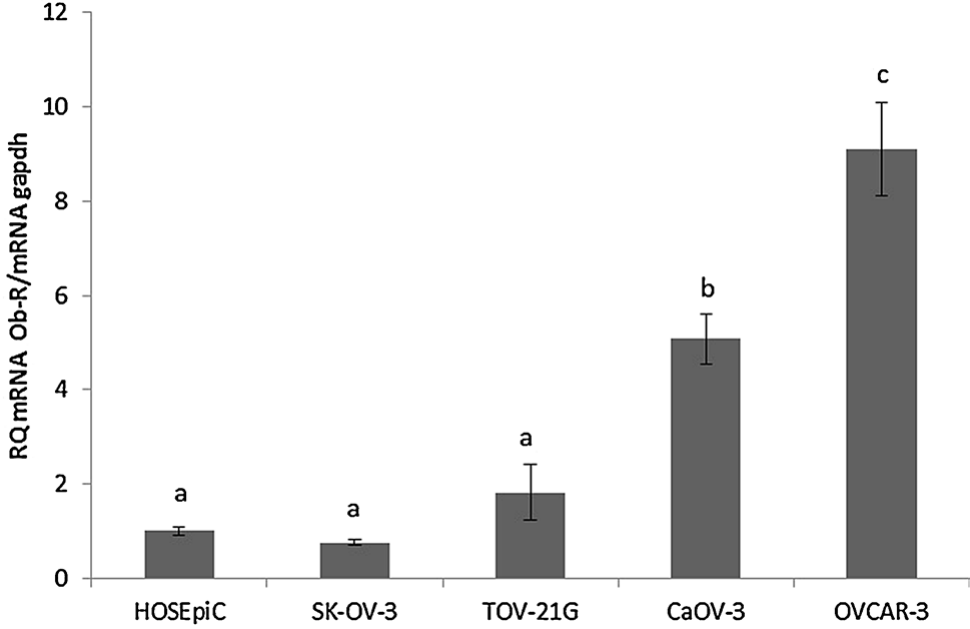
Leptin receptor (ObR) gene expression in different cell lines. Basal mRNA for leptin receptor was evaluated by qPCR after 24 h of cell culture. All the result were normalised to HOSEpiC, value equal to 1. Values are mean ± SEM. Statistically significant differences between cell lines in graph are indicated with different letters; the same letters indicating no significant differences, with a < b < c

The expression of the leptin receptor gene varies between different cell lines. Assuming the expression of the gene for the leptin receptor in non-cancerous human ovarian epithelial cell line (HOSEpiC) to be 1, we noted that in the SK-OV-3 cell line ObR gene expression was comparable to that in HOSEpiC and TOV-21G. A fivefold higher expression of ObR was noted in CaOV-3 and a ninefold higher in the OVCAR-3 cell line (Fig. 1).

### The effect of Leptin on the expression of the leptin receptor (ObR) gene in different cell lines

**Fig. 2:**
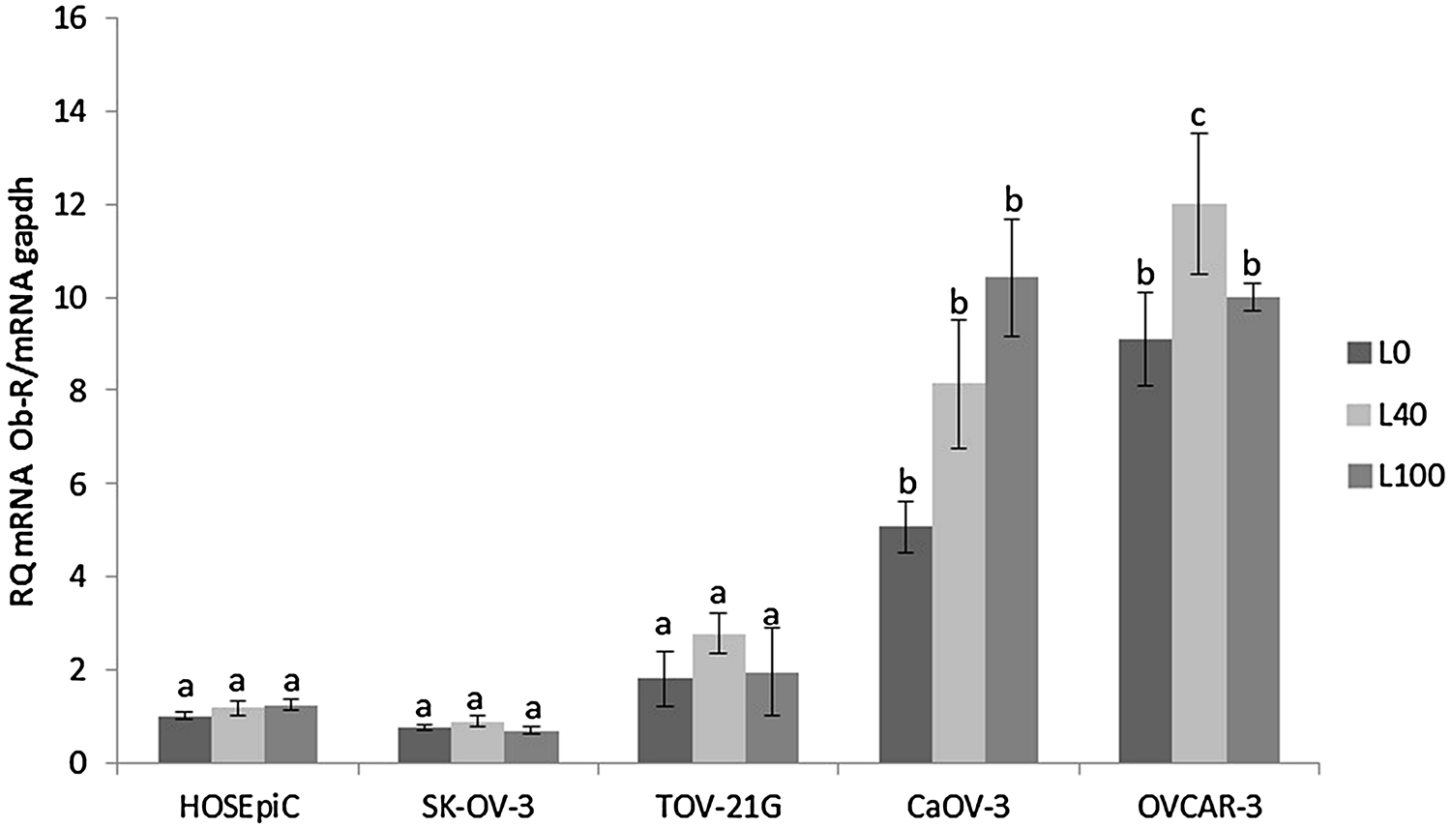
Effect of leptin on the expression of leptin receptor (ObR) gene in different cell lines. Each point represents the mean ± SEM from three independent experiments. Statistically significant different from control are indicated with *different letters*; the *same letters* indicating no significant differences, with a < b < c

Leptin at both doses, 40 and 100 ng/mL, significantly increased ObR gene expression in CaOV-3, while in OVCAR-3 only a dose of 40 ng/mL had an effect (Fig. 2). In the other investigated cell lines, no effect on ObR gene expression was observed. Based on these two experiments, we chose CaOV-3 and OVCAR-3, characterised by the highest expression of mRNA for leptin receptors and response to leptin supplementation, for further research.

### The action of leptin receptor antagonists (SHLA and Lan-2) on ObR protein level

**Fig. 3:**
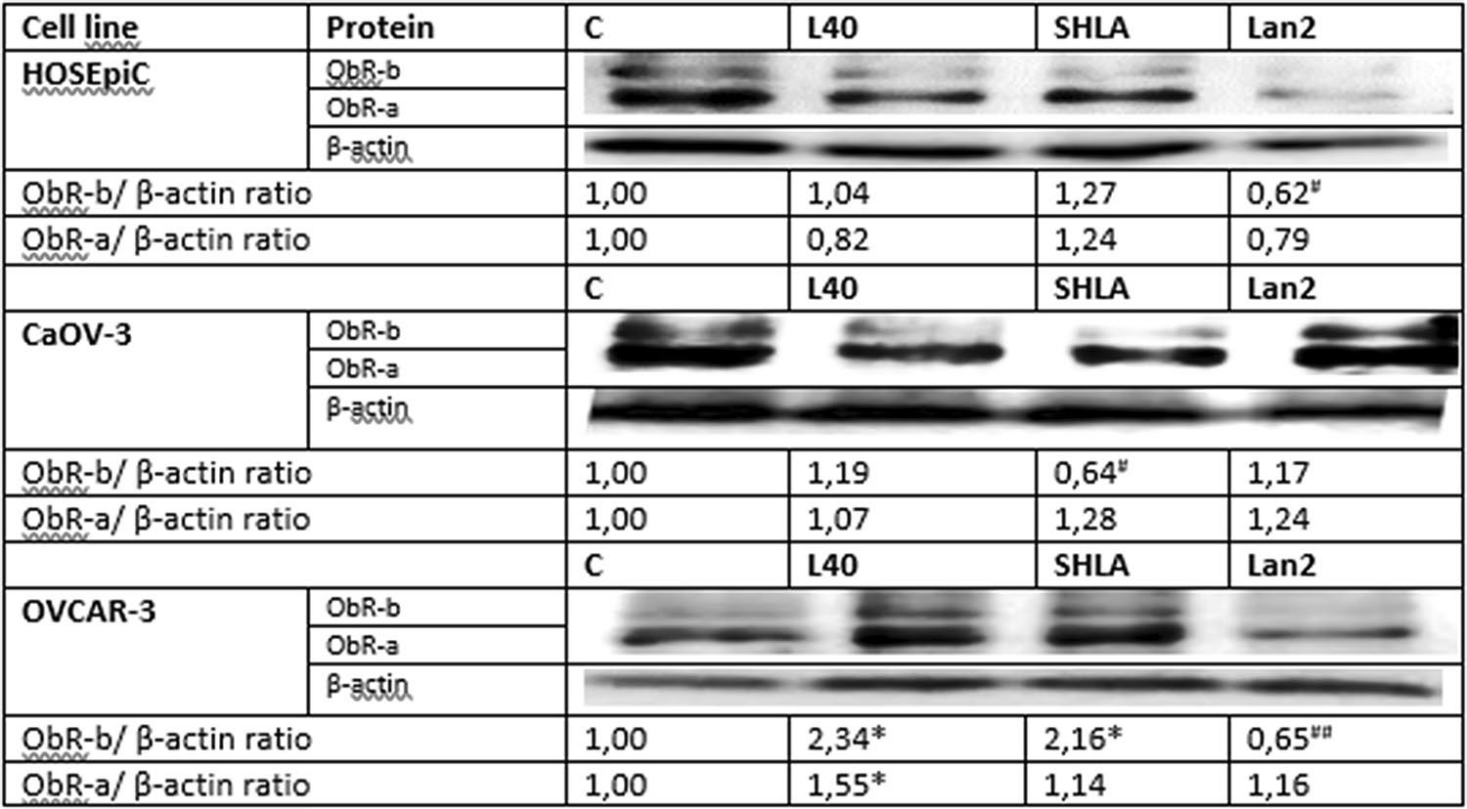
Changes in ObR-a (100 kDa) and ObR-b (125 kDa) protein expression in HOSEpiC, CaOV-3 and OVCAR-3 cells exposed to leptin at dose 40 ng/mL and SHLA and Lan-2 at dose 1000 ng/mL with leptin at dose 40 ng/mL. Control value = 1.0. Densitometry results were normalised to β-actin loading controls to obtain a ratio bands. All values marked with *(*p* < 0.05) are significantly different from control. All values marked with #(*p* < 0.05), ##(*p* < 0.01) are significantly different from values of leptin at dose 40 ng/mL

Using Western blot analysis, we observed that leptin increase both short and long form of leptin receptor (ObR-b) expression only in OVCAR-3 cell line (Fig. 3). From ObR antagonists, Lan-2 reversed leptin action on ObR-b expression to control in HOSEpiC and OVCAR-3 cells, while SHLA in CaOV-3 cells (Fig. 3).

### The action of leptin and leptin receptor antagonists (SHLA and Lan-2) on cell proliferation (Alamar Blue assay, BrdU assay)

**Fig. 4:**
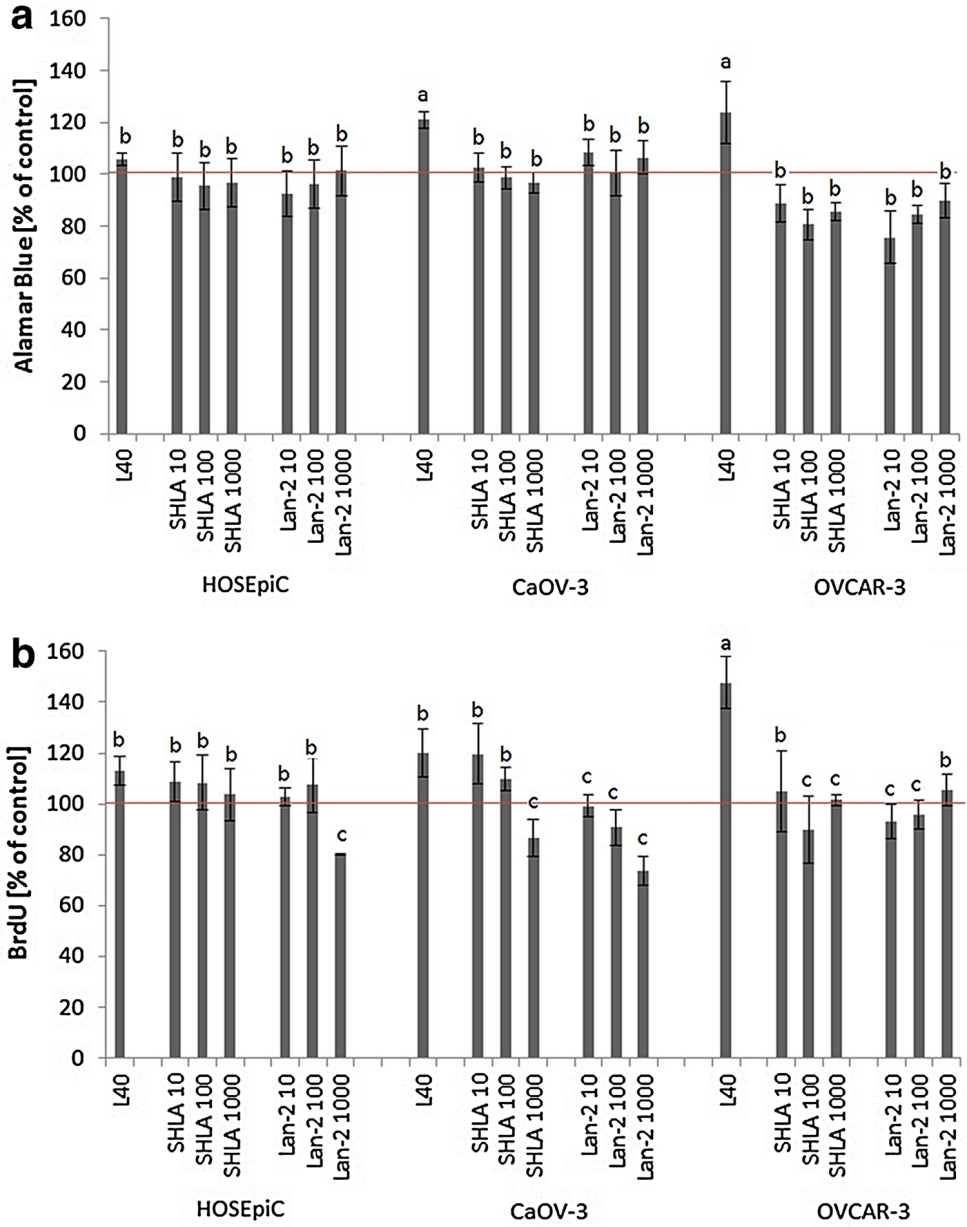
Leptin and leptin receptor antagonists (SHLA and Lan-2) action on HOSEpiC, CaOV-3 and OVCAR-3 cells proliferation measured by **a** Alamar Blue and **b** BrdU incorporation assay. Cells were treated for 48 h with leptin at dose 40 ng/mL alone or with antagonists at doses 10; 100 and 1000 ng/mL. Each point represent the mean ± SEM. from three independent experiments of four replicates per treatment group. Statistically significant different from control are indicated with *different letters*; the *same letters* mean no significant differences, with a < b < c

Leptin at dose of 40 ng/mL (noted in plasma of overweigh human) had a stimulatory effect on both cancer cell lines proliferation (Fig. 4a, b).

Measuring cell proliferation using the Alamar Blue test we noted no effect of both receptor blockers on non-cancerous HOSEpiC cell line proliferation; however, both receptor blockers reversed the stimulatory effect of leptin in CaOV-3 cell line to control levels and below control levels in the OVCAR-3 cell line (Fig. 4a).

Using a BrdU incorporation assay, we noted that Lan-2 at the highest dose (1000 ng/mL) reduces proliferation to below the level found in the control HOSEpiC cells. In the CaOV-3 cell line SHLA at a dose of 1000 ng/mL and Lan-2 at all doses reversed the stimulatory effect of leptin, and in the OVCAR-3 cell line both antagonists at all doses decreased leptin-stimulated cell proliferation (Fig. 4b).

### The effect of SHLA and Lan-2 on selected gene expression involved in cell cycle regulation in CaOV-3 and OVCAR-3 cells

**Fig. 5:**
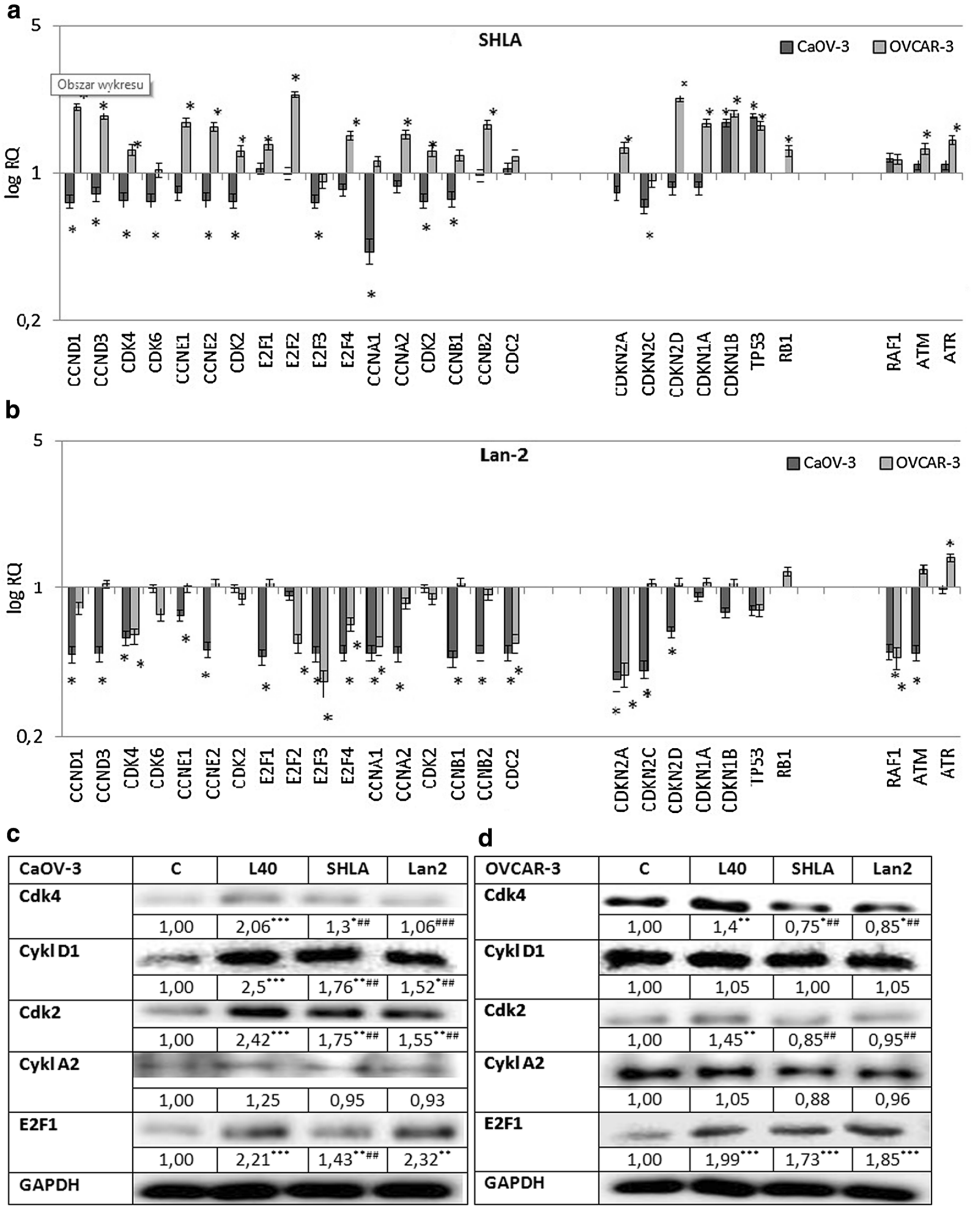
Changes in selected cell cycle genes expression in CaOV-3 and OVCAR-3 cells exposed to SHLA (**a**) and Lan-2 (**b**) at dose 1000 ng/mL with leptin at dose 40 ng/mL for 24 h. All values marked with *(*p* < 0.05) are significantly different from leptin treated (40 ng/mL) control. Control value = 1.0. The effect of SHLA and Lan-2 at dose 1000 ng/mL with leptin at dose 40 ng/mL on cdk2, cyclin A, cdk4, cyclin D and E2F1 protein expression in CaOV-3 (**c**) and OVCAR-3 (**d**) cells. GAPDH was used as a loading control for Western blot analysis. The representative blots of three experiments are shown in the panels. Cdk2, cyclin A, cdk4, cyclin D and E2F1 densitometry results were normalised to GAPDH loading controls to obtain a ratio bands. Values are mean ± SEM All values marked with *(*p* < 0.05), **(*p* < 0.01) and ***(*p* < 0.001) are significantly different from untreated control values. All values marked with #(*p* < 0.05), ##(*p* < 0.01) and ###(*p* < 0.001) are significantly different from values of leptin at dose 40 ng/mL

Based on the results of the experiments concerning the action of leptin receptor blockers on cell proliferation, we chose 1000 ng/ml for the next experiments. SHLA in the CaOV-3 cell line downregulated all investigated cell cycle progression gene expression, with the highest effect on CCNA1 and from cell cycle inhibitors that had a stimulatory action on the CDKN1B and TP53 genes. In the OVCAR-3 cell lines SHLA upregulated both genes involved in cell cycle activation, as well as CDKN2D, CDKN1A, CDKN1B, TP53 cell cycle inhibitors. Additionally, SHLA increased ATM and ATR gene expression, which belong to the PI3/PI4 kinase family and function as regulators of a wide variety of downstream proteins, including tumour suppressor proteins p53 (Fig. 5a).

Lan-2 in CaOV-3 downregulated all investigated genes involved in cell cycle activation and inhibition. Additionally, Lan-2 downregulated RAF1 and ATM gene expression. In OVCAR-3, Lan-2 downregulated genes involved in cell cycle activation, with the highest effect on CDK4, E2F2, E2F3, CCNA1 and CDC2. There was no effect on cell cycle inhibitors except for CDKN2A and p53. Additionally, an inhibitory effect on RAF-1 and a stimulatory effect on ATM and ATR gene expression was noted (Fig. 5b).

### The effect of SHLA and Lan-2 on cdk4, cdk2, cyclin A, cyclin D and E2F1 protein expression in CaOV-3 and OVCAR-3 cells

To investigate action on protein expression, we chose cdk4 and cyclin D from phase G1 of the cell cycle in cdk2 and cyclin A and from phase S, and additionally E2F1, which is a transcription factor with a crucial role in the control of the cell cycle and action on tumour suppressor proteins. In CaOV-3, leptin stimulated the expression of all the investigated proteins except for cyclin A2. SHLA decreased leptin-stimulated expression of cdk4, cyclin D1, cdk2 and E2F1 and had no effect on cyclin A2, while Lan-2 decreased the expression of cdk4, cyclin D1 and cdk2 (Fig. 5c) In OVCAR-3 3 leptin stimulates the expression of cdk4, cdk2 and E2F1. Both SHLA and Lan-2 decreased the leptin-stimulated protein expression of cdk4 and cdk2. This suggests that both antagonists act mainly on cdk/cyclin complexes (Fig. 5d).

### The effect of SHLA and Lan-2 on signalling protein expression in CaOV-3 and OVCAR-3 cells

**Fig. 6:**
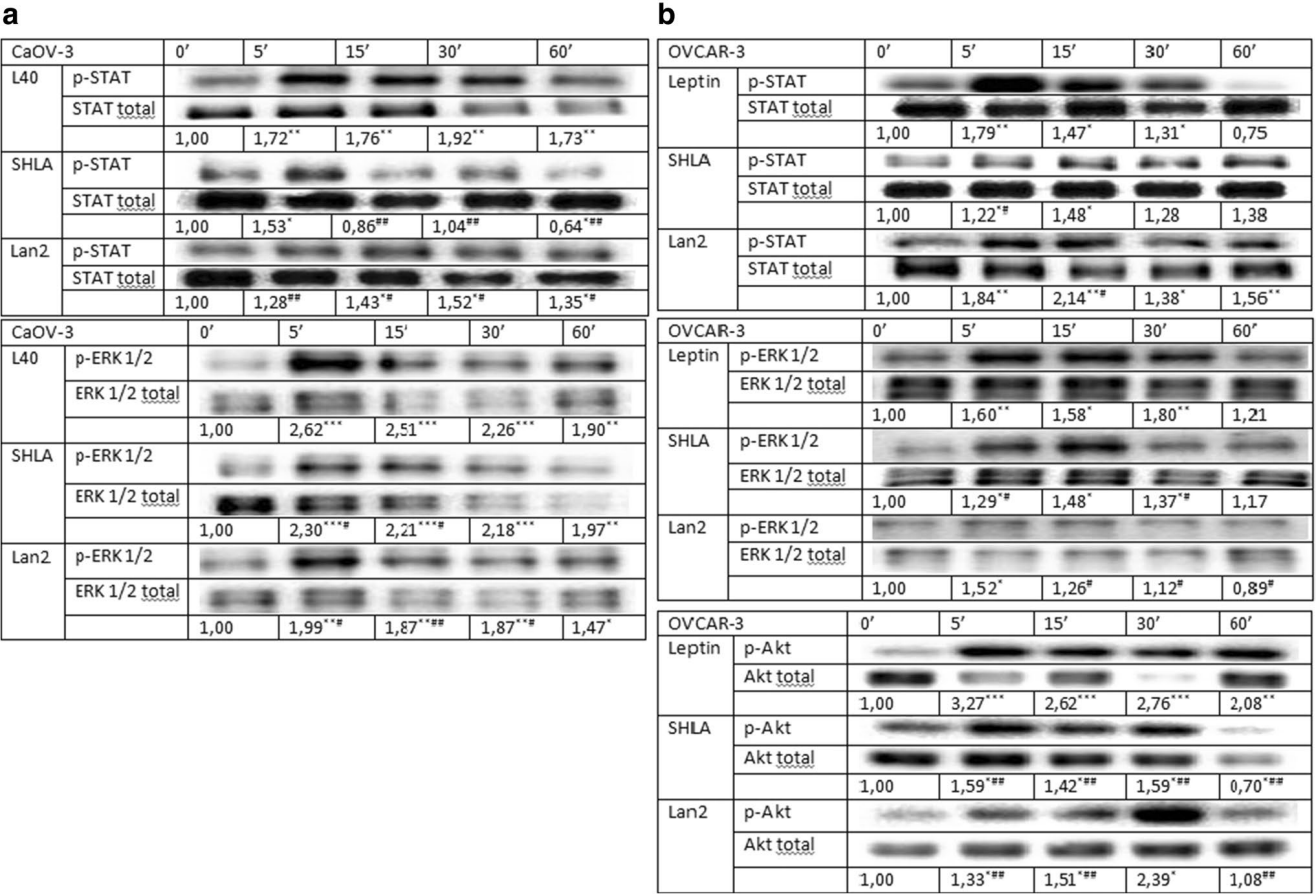
Effect of SHLA and Lan-2 at dose 1000 ng/mL on phospho- and total: STAT, ERK1/2 in CaOV-3 (**a**) and on STAT, AKT, ERK1/2 in OVCAR-3 (**b**) cells. Blots are representative of three experiments. The results of densitometry analyses are expressed as phospho-protein band density normalised to the density of the total form (loading control) bands. All values marked with *(*p* < 0.05), **(*p* < 0.01) and ***(*p* < 0.001) are significantly different from control values. All values marked with #(*p* < 0.05), ##(*p* < 0.01) and ###(*p* < 0.001) are significantly different from values of leptin at dose 40 ng/mL

To investigate the effect of SHLA and Lan-2 on cell signalling pathways activated by leptin we chose three signalling proteins from different pathways: STAT, ERK1/2 and Akt. In the CaOV-3 cell line, both SHLA and Lan-2 inhibited the phosphorylation of the STAT protein, and only Lan-2 decreased the phosphorylation of the ERK1/2 protein (Fig. 6a). Interestingly we did not observe the phosphorylation of the Akt protein in CaOV-3 cells (data not shown). In the OVCAR-3 cell line, we noted that SHLA inhibited phosphorylation in all the investigated proteins and that Lan-2 decreased only Akt phosphorylation (Fig. 6b).

## Discussion

Our data clearly show differences in the expression of the leptin receptor gene depending on histopathological types. Interestingly, in the SK-OV-3 cell line, which is TNF-resistant adenocarcinoma, and TOV-21G, which is clear-cell adenocarcinoma, ObR gene expression was comparable to that seen in non-cancerous HOSEpiC. A fivefold higher expression of ObR was seen in primary ovarian cancer CaOV-3 and a ninefold higher in serous carcinoma OVCAR-3 cells derived from a tumour resistant to chemotherapy. Additionally, we found that only in these two cell lines, CaOV-3 and OVCAR-3, leptin increases its own receptors gene expression. However, only in OVCAR-3 leptin increased both short and long form of leptin receptor (ObR-b) protein expression. Di Yorio et al. [20] using rat ovarian explants culture described that leptin produced an increase in Ob-R expression in concentration-dependent manner with no effect at doses 30 and 100 ng/mL which is in coincidence with our results on non-cancerous HOSEpiC cell line.

These data show differences in the expression of the leptin receptor gene depending on different histopathological ovarian cancer types. Differences in the expression of the short (ObR-a) and long (ObR-b) isoform in various endometrial cancer cells have been described by Gao et al. [21]. Using breast cancer explant collected from patients, Ishikawa et al. [22] showed that among 76 cases of breast cancer only 13 were negative for leptin receptor expression and patients with ObR expression were in the higher-risk group for future survival. In addition, studies conducted on ovarian tumour explants indicated a worse prognosis among patients with a higher expression of the leptin receptor [3].

Considering leptin action on cell proliferation, we noted dose-dependent action in CaOV-3 and OVCAR-3, as well as in the non-cancerous cell line HOSEpiC. These results were confirmed by two independent tests (Alamar Blue, BrdU). The effect of leptin on proliferation has been well investigated in various cell types. Research carried out by our team demonstrated that leptin can stimulate OVCAR-3 cell growth at doses of 2, 20, 40 and 100 ng/mL after 48 and 72 h of treatment [2]. In addition, the stimulatory effect of leptin at a dose of 100 ng/mL on cell proliferation in another ovarian cancer cell line, BG-1, was presented by Choi et al. [1]. The stimulatory effect of leptin on the proliferation of different breast cancer cells, T47-D [11], MCF7 and Zr-75-1 [10, 23–25] has also been examined. Furthermore, it has been found that leptin acts as a proliferation-stimulating factor in prostate, oesophageal [10], liver [26] and colon cancer [27]. Interestingly, Somasundar et al. [10] have shown that leptin can inhibit the proliferation of pancreas cancer cells at a dose of 0.4 and 4 ng/mL.

The main aim of this study was to determine the possible use of the leptin receptor blockers superactive human leptin antagonist (SHLA) and quadruple leptin mutein, Lan-2 (L39A/D40A/F41A/I42A), as additional therapy factors for the treatment of ovarian cancer. From ObR antagonists Lan-2 reversed leptin action on ObR-b expression to control in HOSEpiC and OVCAR-3 cells, while SHLA in CaOV-3 cell.

No effect of either receptor blocker on non-cancerous HOSEpiC cell line proliferation; however, both receptor blockers reversed the stimulatory effect of leptin on CaOV-3 cell line proliferation to control levels and to below control levels in the OVCAR-3 cell line. To our knowledge, these are the first data that have shown the inhibition of leptin-stimulated proliferation in ovarian cancer and non-cancerous cells under the treatment of leptin receptor antagonists. These blockers have not, until now, been used in investigations of ovarian cell proliferation. Of the two, only SHLA has been examined on OE33 (oesophageal adenocarcinoma cells) alone or in combination with cisplatin by Bain et al. [18] and been shown to inhibit proliferation. Using the ObR antagonists Aca-1 and Allo-aca Otvos et al. [15, 28] demonstrated that the leptin-stimulated proliferation of MCF-7 and MDA-MB-231 breast cancer cells (receptively) can be inhibited in a dose-dependent manner. Beccari et al. [29] tested the ObR antagonists Allo-aca, D-Ser, DDD and others, showing that the majority of tested leptin receptor antagonists reverse the leptin-stimulated proliferation of human breast cancer cells (MCF-7) and human colon adenocarcinoma cells (HT29). Catalano et al. [16] described reversed leptin-stimulated proliferation to control levels in the breast cancer cells MCF-3 and SKBR-3 in a dose-dependent manner using another LDFI ObR antagonists. In addition, Scolaro et al. [30] showed a dose-dependent effect of Allo-aca on the cell proliferation of RF/6A (monkey endothelial retinal cells) and BCE (bovine endothelial corneal cells).

Going inside the mechanism of action, we showed that in the CaOV-3 cell line both SHLA and Lan-2 downregulate all investigated cell cycle progression of gene expression and that cell cycle inhibitors had a stimulatory effect on CDKN1B and TP53 and an inhibitory effect on CDKN2C. From the selected protein expression, both SHLA and Lan-2 decreased the leptin-stimulated expression of cdk4, cyclin D1 and cdk2, and SHLA additionally affected E2F1 and had no effect on cyclin A2. In OVCAR-3, only Lan-2 downregulated genes involved in cell cycle activation and had an inhibitory effect on RAF-1 and a stimulatory effect on ATM and ATR gene expression. Both SHLA and Lan-2 decreased leptin-stimulated protein expression by cdk4 and cdk2 and had no effect on cyclin D1 and A2. The impact of leptin on cell cycle gene and protein expression in different cancer cells is well established. In OVCAR-3 ovarian cancer cells, Chen et al. [31] have shown that leptin at a dose of 50 ng/mL can stimulate the expression of cyclin D1. In the same year, Ptak et al. [2] demonstrated similar changes in cyclin D1 expression in the OVCAR-3 cell line under the influence of 40 ng/mL of leptin and additionally on cyclin A expression. A stimulatory effect on cyclin D1 protein expression was also shown in breast cancer [31, 32], endometrial cancer [33] and colon cancer cells [34] and in hepatocellular carcinoma [35].

To our knowledge, there are no data concerning the action of ObR antagonists on cell cycle gene expression. Our data clearly show different effects depending on cancer cell types. In chemoresistant OVCAR-3 cells, SHLA had no inhibitory effect on cell cycle progression gene expression and decreased only cdk2 and cdk4 protein expression without having an effect on cyclin A2 and D1. In CaOV-3, both ObR blockers down regulated the cell cycle progression gene and, apart from cdk2 and cdk4, cyclinD protein expression. Interestingly, our data showed a lack of an effect by both ObR antagonists on E2F1 in OVCAR-3 cells, but downregulation in CaOV-3 cells under the influence of SHLA. Reimer et al. [36] suggested that the deregulation of E2F factors is one of the most important events in the chemotherapy resistance formation of ovarian cancer, confirming that OVCAR-3 cells are chemoresistant. E2F1 factor, which is a transcription activator promoting the cell cycle, is overexpressed in epithelial ovarian cancers (EOC) and is associated with higher stage and tumour grade [37].

In spite of the statement by Masamha et al. [38], who, based on an experiment on A2780 and SK-OV-3 cells, showed that cyclin D1 inhibition is necessary to inhibit cell cancer growth and is a potential therapeutic target in ovarian cancer cells, we declare that both ObR blockers, by downregulating cdk2 and cdk 4, can downregulate the formation of the cyclin A/cdk2 and cyclin D/cdk4 complex and inhibit cell proliferation. In addition, the direct impact on cell cycle gene expression action on cycle proliferation can be the result of the effect on signalling pathways.

Leptin is able to activate several signalling pathways, such as JAK/Stat3, MAPK/ERK and PI3 K/Akt, in various cell types. In ovarian cancer, it has been demonstrated by Chen et al. [13] that leptin activates Akt and ERK1/2 proteins in the OVCAR-3 cell line. Uddin et al. [8] showed that leptin stimulates the activation of the PI3 K/Akt pathway in MDAH2774 and SK-OV-3 ovarian cancer cells. In our data, we have shown that leptin stimulates the phosphorylation of Stat3, ERK1/2 and Akt. [2]. The activation of the mentioned pathways by leptin has also been described in endometrial [21], prostate [39], liver [32] and colon cancer cells [40].

As a second messenger system involved in possible action on leptin receptor antagonists, we investigated the effect on the JAK/Stat3, MAPK/ERK and PI3 K/Akt pathways. In OVCAR-3 cells, SHLA inhibited the phosphorylation of all tested signalling proteins, which was correlated with inhibitory effect on leptin-stimulated cell proliferation. Lan-2 acted only on the Akt phosphorylation and, however, additionally downregulated ObR-b protein expression. We suggest that a stronger inhibition of proliferation observed under the influence of Lan-2 is a consequence of the downregulation ObR-b. In CaOV-3, Lan-2 inhibited Stat3 and ERK1/2 proteins and in the consequence decreased leptin-stimulated cell proliferation. SHLA only inhibited Stat3 and, however, in opposite to OVCAR-3 additionally downregulated ObR-b protein expression in CaOV-3. These results agree with the limited available data on ObR antagonist actions on cell signalling. The Lan-1 antagonist, which is similar to Lan-2, effectively inhibited leptin-induced phosphorylation of Jak2, Akt and ERK1/2 in a dose-dependent manner in the LNCaP prostate cancer cell line (Samuel–Mendelhson et al. 2011). In addition, Otvos et al. (2011) have shown that Aca1 can inhibit Stat3 phosphorylation in MCF-7 cells. In MCF-7 and SKBR3 breast cancer cells, pre-treatment with a LDFI-leptin receptor antagonist abrogated the leptin activation of Jak2, Stat3, Akt and ERK1/2 proteins [16].

In summary: (1) In metastatic carcinoma CaOV-3 both ObR antagonists had an inhibitory effect on the cdk2/cyclin D1 complex, while in serous carcinoma, OVCAR-3, they had an effect only on cdk2 and cdk4 protein expression, (2) SHLA had an inhibitory effect on all the investigated signalling pathways in OVCAR-3, but only on Stat3 in CaOV-3, (3) Lan-2 had an inhibitory effect on Stat3 and ERK1/2 CaOV-3, while in OVCAR-3 it only had an effect on Akt protein phosphorylation.

In conclusion, to our knowledge this study is the first to show the possible use of leptin receptor antagonists in blocking the proliferation and progression of epithelial ovarian tumours. Based on the results we suggest that SHLA and Lan-2 are promising leptin receptor inhibitors that could be used to block leptin activity, eliminating its negative effects on activities related to ovarian carcinogenesis. However, selection of an antagonist for use should be related to tumour type. Future studies should be carried out using explants from patients with ovarian cancer and then validated in in vivo models, which would allow the investigation of the effects of leptin receptor antagonists on living organisms.
